# Pediatric resident and faculty attitudes toward self-assessment and self-directed learning: a cross-sectional study

**DOI:** 10.1186/1472-6920-9-16

**Published:** 2009-04-13

**Authors:** Su-Ting T Li, Michele A Favreau, Daniel C West

**Affiliations:** 1 Department of Pediatrics, University of California Davis, Sacramento, California, USA; 2 Office of the Dean, University of California Davis, Sacramento, California, USA; 3 Department of Pediatrics, University of California San Francisco, San Francisco, California, USA

## Abstract

**Background:**

The development of self-assessment and self-directed learning skills is essential to lifelong learning and becoming an effective physician. Pediatric residents in the United States are now required to use Individualized Learning Plans (ILPs) to document self-assessment and self-directed learning. A better understanding of resident and faculty attitudes and skills about self-assessment and self-directed learning will allow more successful integration of lifelong learning into residency education. The objective of this study was to compare faculty and resident attitudes, knowledge and skills about self-assessment, self-directed learning and ILPs.

**Methods:**

Survey of pediatric residents and faculty at a single institution. Respondents rated their attitudes, knowledge, and self-perceived skills surrounding self-assessment, self-directed learning and ILPs.

**Results:**

Overall survey response rate was 81% (79/97); 100% (36/36) residents and 70% (43/61) faculty. Residents and faculty agreed that lifelong learning is a necessary part of being a physician. Both groups were comfortable with assessing their own strengths and weaknesses and developing specific goals to improve their own performance. However, residents were less likely than faculty to continuously assess their own performance (44% vs. 81%; p < 0.001) or continuously direct their own learning (53% vs. 86%; p < 0.001). Residents were more likely than faculty to believe that residents should be primarily responsible for directing their own learning (64% vs. 19%; p < 0.0001), but at the same time, more residents believed that assigned clinical (31% vs. 0%; p < 0.0001) or curricular (31% vs. 0%; p < 0.0001) experiences were sufficient to make them competent physicians. Interns were less likely than senior residents to have a good understanding of how to assess their own skills (8% vs. 58%; p = 0.004) or what it means to be a self-directed learner (50% vs. 83%; p = 0.04).

Qualitative comments indicated that while ILPs have the potential to help learners develop individualized, goal-directed learning plans based on strengths and weaknesses, successful implementation will require dedicated time and resident and faculty development.

**Conclusion:**

These findings suggest that training and experience are necessary for physicians to understand the role of self-directed learning in education. Deliberate practice, for example by requiring residents to use ILPs, may facilitate self-directed, lifelong learning.

## Background

There is broad consensus that the development of self-assessment and self-directed learning skills is essential to lifelong learning and a critical step towards becoming an effective physician[1]. Lifelong learning is considered by many to be an integral component of professionalism [2-4]. In 2002, the American Board of Internal Medicine Foundation, the American College of Physicians Foundation, and the European Federation of Internal Medicine published a physician charter defining medical professionalism for the new millennium, which included the statement that "physicians must be committed to lifelong learning"[2,3]. This charter was endorsed by over 120 national and international organizations across the world and was translated into 10 languages[4,5]. Documentation of lifelong learning is now required for residency training, board certification and maintenance of certification in the United States, Canada, Australia, New Zealand and many European countries [6-8]. Furthermore, in the United States, the Review Committee for pediatrics of the Accreditation Council for Graduate Medical Education now requires all training programs to use Individualized Learning Plans (ILPs) to document resident self-assessment and self-directed learning as part of the practice-based learning and improvement competency[9].

Despite the importance of self-assessment to lifelong learning, previous studies have shown that physicians have limited ability to self-assess[10]. In addition, those who were the least skilled and the most overconfident were also the least accurate when self-assessment was compared with external assessment (e.g. faculty evaluations, multisource feedback, observed interactions, written examinations) [10-12]. Physicians' limited ability to self-assess makes development of these skills during training important. However, relatively little is known about how best to teach the components of lifelong learning: self-assessment and self-directed learning. Even less is known about how ILPs, now required for pediatric residents in the United States, can help in that process.

Developing a better understanding of how to teach and learn these self-assessment and self-directed learning skills is important, because producing physicians that are better lifelong learners may improve the quality of care that patients receive. To begin to address this issue, our study sought to understand resident and faculty attitudes, knowledge, and skills surrounding self-assessment, self-directed learning and ILPs prior to implementation of ILPs in our pediatric residency program. This is important because successful implementation of ILPs depends on (1) understanding baseline attitudes, knowledge, and skills of residents and faculty toward self-assessment and self-directed learning and (2) identifying potential resident and faculty biases toward the overall ILP process. We hypothesized that residents and faculty have different attitudes and skills related to self-assessment and self-directed learning. Developing a better understanding of these differences may allow for more effective integration of lifelong learning strategies into residency education and ultimately result in improved lifelong learning in practicing physicians.

## Methods

This study was performed prior to the introduction of ILPs in our program. A paper-based survey was distributed to all pediatric residents in mid-July 2007. A web-based survey was sent to all pediatric faculty in late July 2007. Reminder emails were periodically sent out until the survey closed in August 2007.

The survey asked respondents to rate their attitudes, knowledge, and self-perceived skills surrounding self-assessment, self-directed learning, and ILPs on a 6-point Likert scale, with 1 indicating strongly disagree and 6 indicating strongly agree. In addition, residents were asked their gender, year of residency, area of pediatrics they were interested in (general pediatrics, subspecialty, undecided), and future intended practice setting (academic, community, undecided). Faculty was asked whether they were currently an advisor for a resident, their gender, their area of pediatrics (general pediatrics, subspecialty), number of years ago they completed fellowship/residency training, and distribution of their time (clinical, teaching (not clinical), research, administration).

The University of California, Davis Institutional Review Board approved this study.

### Quantitative analysis

In all survey questions, the 6 point Likert scale was dichotomized by collapsing the strongly agree and agree responses into one category and all other responses into a second category. Univariate analysis was used to describe the self-perceived attitudes, knowledge, and skills about self-assessment and self-directed learning. Bivariate analyses were performed using Pearson's chi-square. Our primary analysis compared resident and faculty agreement (strongly agree, agree) with the statements in the survey. In order to explore differences based on level of training, a sub analysis was performed comparing first-year resident responses to senior (second- and third-year) resident responses.

All statistical analysis was conducted using STATA 8.0 statistical software[13].

### Qualitative analysis

Two authors (STL and MAF) reviewed the free text responses on the survey and independently identified themes. Data were collected as part of a purposeful sampling and manually coded into categories using inductive analyses. Coding discrepancies of the identified themes were resolved by consensus discussion.

## Results

The overall response rate was 81% (79/98), 100% (36/36) of residents and 70% (43/61) of faculty. A total of 12 first-year residents and 24 senior residents (12 second-year and 12 third-year residents) responded. Table 1 displays the demographic data of the respondents and all faculty. Faculty respondents and non-respondents were similar in gender, specialty area, and whether or not they were currently faculty advisors. Compared with residents, faculty was more likely to be male, a subspecialist (or planning subspecialty training), and practice in an academic setting (or planning to practice in an academic setting).

**Table 1:** Demographics of respondents

	**Residents**; N = 36	Faculty*; N = 43	**All Faculty **(Both Respondents and Nonrespondents); N = 61
**Male**	6 (18%)	22 (61%)	33 (54%)

**Specialty area**			
General pediatrics	18 (51%)	10 (28%)	15 (25%)
Subspecialty	8 (23%)	26 (72%)	46 (75%)
Undecided	9 (26%)		

**Practice setting**			
Community	11 (31%)		
Academic	5 (14%)	100%	100%
Undecided	19 (54%)		

**Faculty advisor for resident**		20 (47%)	27 (44%)

**Number of years since completion of training**			
0–5		7 (20%)	NA
6–10		11 (31%)	NA
11–15		3 (9%)	NA
16 or more		14 (40%)	NA

**How is your time spent?**			
Clinical		51%	NA
Teaching (not clinical)		9%	NA
Research		26%	NA
Administration		18%	NA

*Not all participants completed the demographic questions. NA = not available.

Table 2 compares resident and faculty responses. Residents and faculty responses were similar in many areas. Both groups agreed that lifelong learning is necessary to being a physician. They also agreed that self-assessment and self-directed learning improves patient care. Residents and faculty were equally comfortable assessing their own areas of strengths and areas for improvement and developing specific goals to improve their own performance. Residents were less likely than faculty to continuously assess their own performance (44% vs. 81%; p < 0.001) or continuously direct their own learning (53% vs. 86%; p < 0.001).

**Table 2:** Comparison of resident and faculty attitudes toward self-assessment and self-directed learning

**Item**	**Resident; **No. (%) Agree/Strongly Agree	**Faculty; **No. (%) Agree/Strongly Agree	**p-value**
1. I have a good understanding of how to assess my own skills.	15 (42%)	21 (49%)	0.52

2. I have a good understanding of what it means to be a self-directed learner.	26 (72%)	37 (86%)	0.13

3. I have a good understanding of what an Individualized Learning Plan (ILP) is.	16 (44%)	14 (33%)	0.28

4. I am confident in my ability to write an effective ILP to improve my performance. (Faculty: I am confident in my ability to help my resident write an effective ILP to improve his/her performance.)	9 (25%)	12 (28%)	0.77

5. I am confident in my ability to assess my own performance.	14 (39%)	26 (60%)	0.06

6. I am confident in my ability to identify my strengths.	16 (44%)	26 (60%)	0.16

7. I am confident in my ability to identify my areas for improvement.	19 (53%)	29 (67%)	0.18

8. I am confident in my ability to write specific goals to improve my performance.	17 (47%)	21 (51%)	0.73

**9. I continuously assess my own performance.**	**16 (44%)**	**35 (81%)**	**<0.001**

**10. I continuously direct my own learning.**	**19 (53%)**	**36 (86%)**	**<0.001**

11. Lifelong learning is necessary to being a physician.	33 (92%)	42 (98%)	0.23

**12. Residents should be primarily responsible for directing their own learning.**	**23 (64%)**	**8 (19%)**	**<0.001**

13. Faculty should be primarily responsible for directing residents' learning.	9 (25%)	9 (21%)	0.71

14. I believe that physician self-assessment improves patient care.	28 (78%)	31 (74%)	0.68

15. I believe that physician self-directed learning improves patient care.	31 (86%)	35 (83%)	0.74

**16. My assigned clinical experiences (rotations, clinics) are sufficient to make me a competent physician. (Faculty: Resident's assigned clinical experiences (rotations, clinics) are sufficient to make him/her a competent physician.)**	**11 (31%)**	**0 (0%)**	**<0.0001**

**17. My assigned curricular experiences (lectures, didactics, readings) are sufficient to make me a competent physician. (Faculty: Resident's assigned curricular experiences (lectures, didactics, readings) are sufficient to make him/her a competent physician.)**	**11 (31%)**	**0 (0%)**	**<0.0001**

Residents were more likely than faculty to feel that residents should be primarily responsible for directing their own learning (64% vs. 19%; p < 0.0001). Residents were also more likely to believe that assigned clinical experiences (rotations, clinics) (31% vs. 0%; p < 0.0001) or assigned curricular experiences (lectures, didactics, readings) (31% vs. 0%; p < 0.0001) were sufficient to make residents competent physicians.

Residents and faculty were more confident in their ability to identify their individual strengths and weaknesses (44–65%) and write specific goals to improve their own performance (47–51%), but were less confident in their ability to write an effective ILP to improve their own performance (25–28%).

### First-year vs. senior residents

When first-year residents (n = 12) were compared to senior (second- and third-year) residents (n = 24), both groups believed that lifelong learning is necessary to being a physician. In addition, both first-year and senior residents believed that residents should be primarily responsible for directing their own learning and that assigned clinical (33% vs. 29%; p = 0.80) and curricular (42% vs. 25%; p = 0.31) experiences were sufficient to make them competent physicians. However, first-year residents were less likely to have a good understanding of how to assess their own skills (8% vs. 43%; p = 0.004) or what it means to be a self-directed learner (50% vs. 83%; p = 0.0.04). In addition, first-year residents were less confident in their ability to assess their own performance (8% vs. 54%; p = 0.008). While interns trended toward being less confident in their ability to write specific goals to improve their performance (25% vs. 58%; p = 0.20), the difference was not statistically significant. First-year residents were less likely than senior residents to continuously assess their own performance (17% vs. 53%; p = 0.02), but were equally likely to continuously direct their own learning (42% vs. 53%; p = 0.35).

### Qualitative results

Table 3 shows that residents and faculty identified several similar advantages to developing an ILP. These included: focused, goal-directed learning plan, "focus, will actually have goals," learning plan based on improvement through identification of learner strengths and weaknesses, "it allows you to pinpoint your areas of weakness and strength and ... create a plan to address your weaknesses," individualized learning, "it will be targeted to the individual's style of learning ..." and accountability, "more accountability." Some faculty additionally commented on the potential benefit of ILPs to faculty through: "focused expectations for resident and instructor," and "identifying areas of strength and of goals for improvement in collaboration between residents and faculty."

**Table 3:** Qualitative Results: Advantages to developing ILPs

**Resident comments**	**Faculty comments**
**Focused, goal-directed learning plan**	
• Focus. Will actually have goals.	• Make learning goals explicit, specific, and do-able.
• Goal-oriented strategy to bring to my practice.	• Focused expectations for resident and instructor.
• Individualized goals and milestones to reach a unique and individualized end point.	
**Learning plan based on improvement through identification of personal strengths and weaknesses**	
• It allows you to pinpoint your areas of weakness and strength and in turn create a plan to address your weaknesses.	• Identifying areas of strength and of goals for improvement in collaboration between residents and faculty.
	• An ILP provides structure and support to the resident to help them improve on their areas of weakness to become better overall physicians.
	• You know your weaknesses and can address these areas.
**Individualized learning**	
• It can be tailored to my learning style and learning needs.	• It will be targeting to the individual's style of learning and future career needs.
• Directed to my interest.	• Helps already educated physicians tailor their ongoing education.
**Accountability**	
• Accountability of knowledge.	• Forces the resident to self-assess and to develop ability and hopefully commit to self-improvement.
• Forces residents to stay on track in reading.	• Forced to reflect on strengths and weaknesses, forced to reflect on goals, forced to reflect on strengthening strengths and improving weaknesses.
• More accountability.	• Defined times for forced self-assessment.
**Self-directed learning**	
• Self-directed	• Communicating the idea that the resident is primarily responsible for his/her own education.
	• Self-directed learning. Places more responsibility for learning on resident.
	• Self-assessment and self-directed goal setting are built into the ILP.

In addition, Table 4 shows that both residents and faculty identified similar areas of challenge. Both groups expressed concern over the amount of time involved to "develop" and "implement" the plan. Residents expressed concern over "finding time in our schedule to follow our plan" and how this time could be better utilized, as it might "take time away from reading." Faculty expressed concern over faculty time to "develop and monitor" the ILP. Another challenge that was identified by both groups involved insufficient understanding of how to construct an effective ILP. This was evidenced by commentary noting a lack of "knowing exactly what to include," and lack of "adequate understanding of strengths/weaknesses and goals that should be achieved." Both groups also remarked on "follow through" as an area of challenge. In addition, residents expressed concern about "maintain [ing] the same goals/plans no matter the rotation," while faculty commented on resident's "motivation to develop it [ILP] and desire to make changes."

**Table 4:** Qualitative Results: Challenges to developing ILPs

**Resident comments**	**Faculty comments**
**Time**	
• Takes time away from reading.	• Finding the time to keep with the program.
• Time-consuming in what is already a limited/full schedule.	• Time to develop and monitor.
**Insufficient understanding of how to construct an effective ILP**	
• Ability to recognize your weaknesses.	• Adequate understanding of strengths/weaknesses and goals that should be achieved.
• Determining one's own strengths and weaknesses and finding ways to utilize and improve them respectively.	• Residents may not have enough insight to develop effective ILPs, and faculty advisors may not be able to a.) spend enough time in helping with the ILP development, and b.) know the resident well enough.
• Knowing exactly what to include.	• Specifically identifying areas of weakness in a resident's education that should be addressed, and how to best structure a plan to adequately provide the resources to address this area.
• Creating a realistic plan that will be easy to follow.	• Knowing what elements work and don't work.
• Knowing what sources to refer to. Setting reasonable goals that can be accomplished while working.	• Faculty need instruction on what is involved.
**Maintaining same goals throughout residency training**	
• We have such a variable schedule that anyone who can maintain the same goals/plan no matter the rotation deserves FIVE GOLD STARS.	
• My interests may change.	
• May be too narrow in scope.	
**Appropriate self-discipline and attitude (for residents)**	
• Finding adequate time and motivation to really focus on it.	• Denial, lack of self-discipline, fatigue.
	• Attitude.
	• Motivation to develop it and desire to make changes.
	• Tunnel vision of most residents. A sense of entitlement and lack of sacrifice.
	• Learning how to make a reasonable effort.
**Follow through**	
• Follow through	• Organization and follow through.
	• I think the most important step in ILP is finding the time to implement the plan once you have identified your learning goals – need to set aside time for timely reading and review of literature, etc.

## Discussion

We found that residents and faculty agreed that lifelong learning is necessary to being an effective physician. However, a majority of residents, but a minority of faculty, believed that residents should be primarily responsible for directing their own learning. At the same time, a third of residents, but no faculty, thought that assigned clinical and curricular experiences were sufficient to make an individual a competent physician. Because these views are inconsistent, and the fact that residents believed this and faculty did not, suggests the possibility that the training received and experience gained during residency training and beyond are important in developing a full understanding of how self-directed learning fits into practice. This finding also underscores the importance of fostering the ongoing development of self-assessment and self-directed learning skills throughout the continuum of medical education.

Only half the residents and faculty had a good understanding of how to assess their own skills or were confident in their ability to do so. In addition, first-year residents were less likely than senior residents to have a good understanding of how to assess their own skills and were less confident in their ability to do so. The challenge of "determining one's own strengths and weaknesses" was also voiced in the qualitative comments, and is consistent with previous studies that have shown that physicians have limited ability to self-assess[10]. Fortunately, previous studies have shown that physician self-assessment skills can be improved through performance feedback[14]. When fourth-year medical students were shown their standardized patient ratings and how their performance compared with that of other students, their self-assessment of their clinical skills improved[14]. In addition, studies of portfolios have indicated that reflection is enhanced with adequate faculty mentorship[15,16]. Our findings, and those of these other studies suggest it would be useful to design ILPs in a way that requires residents to pair their own self-evaluation with external evaluations (such as multisource feedback) with the help of a faculty advisor in order to further develop self-assessment skills.

Our study also showed that residents were less confident in their ability to develop effective ILPs than they were in their ability to assess their own skills and develop specific goals to improve performance. This discrepancy is highlighted in our qualitative data, in which residents describe challenges to developing an ILP that include "creating a realistic plan" to accomplish their specific goals, "adequate time and motivation to really focus on it," and "follow [ing] through with the plan." These comments suggest that residents acknowledge that the challenge of self-directed learning is not just assessing weaknesses and setting specific goals to improve those weaknesses, but developing and implementing realistic plans to accomplish these goals. This commentary suggests that in order for ILPs to be effective, faculty should try to assist learners with the development and implementation of realistic plans.

Residents were less likely than faculty to continuously assess their own performance or continuously direct their own learning even though survey results demonstrated that they felt primarily responsible for their own learning. Our findings support previous reports that suggest that physicians-in-training may not be well-prepared to self-direct their learning[17,18]. For example, when third-year medical students in a family medicine clerkship were given the opportunity to choose their own learning goals, most selected from prewritten learning goals rather than develop their own[17]. In a pilot study of ILPs in a pediatric continuity clinic, most (90%) residents were able to develop learning goals, but few (25%) documented progress toward achieving their goals[18]. Our results and the results of these previous studies suggest the need to develop tools to improve self-directed learning skills. Exposure to problem-based learning curricula[19,20] has been shown to improve self-directed learning in pediatric residents, but other tools to promote self-directed learning, such as ILPs[21], have not been well studied.

Our study has several limitations and identified several potential barriers to implementation of ILPs that must be considered. The survey was administered at a single institution; therefore, generalizability is limited because it remains possible that residents and faculty at other institutions and other specialties may have different attitudes toward physician self-assessment and self-directed learning. However, our institution is similar to other academic medical centers in that faculty have trained in a broad representation of training programs and residents come from a wide range of medical schools throughout the United States. The demographic differences we found between residents and faculty (practice setting, subspecialty, gender) in our study may be a confounding factor, but our study had insufficient power to test this possibility. By definition, all faculty practice in an academic institution, most academic pediatricians are subspecialists, and until recently, there were fewer women in pediatrics (and medicine, in general)[22,23]. A larger study will need to be done in order to more closely examine these factors. Faculty who responded to the survey may be different from faculty who did not respond to the survey. However, demographics of faculty respondents and all faculty were similar. Finally, in our survey we discovered that some faculty and residents did not have a good understanding of what an ILP was. In addition, many residents were unsure how to write an effective ILP and faculty were unsure of their ability to help them. While these findings may be due to the fact that the survey was distributed prior to initiation of ILPs, they underscore the need to provide appropriate training for faculty and residents prior to implementation of an ILP.

Our paper describes initial attitudes, knowledge and skills surrounding self-assessment, self-directed learning and ILPs prior to the implementation of ILPs in our program. Our study suggests that self-assessment and self-directed learning are not innate skills and need to be developed over the course of training. While ILPs may be a tool to improve self-assessment and self-directed learning skills through deliberate practice, our study suggests that several challenges will need to be addressed before ILPs can be successfully implemented. These include: assisting residents and faculty in viewing ILPs as a valuable tool to improve lifelong learning, not merely a "time-consuming" exercise; building flexibility into ILPs in order to meet the evolving goals of learners with different learning styles; and better integrating ILPs into residency training. Ideally, dedicated time would be provided to develop and implement ILPs. Resident and faculty development is needed on how to construct an effective ILP. Such training would most likely involve how to (1) compare external assessment with self-assessment to identify strengths and weaknesses, (2) write specific goals focused on individual improvement, and (3) construct realistic plans to achieve those goals. ILPs could be actively integrated into clinical rotations by having residents share their learning goals with attendings at the beginning of each clinical rotation, so that learning opportunities surrounding specific learning goals could be optimized and learning would become a more "collaborative" partnership.

Further research is needed to better understand how best to integrate ILPs into graduate medical education and whether ILPs help residents develop self-assessment and self-directed learning skills. A repeat survey after implementation of ILPs may help determine whether actual implementation of ILPs affects resident attitudes, knowledge or skills surrounding self-assessment, self-directed learning and ILPs. A national survey of residents at multiple training programs could validate the findings from our study and improve its generalizability. Research is needed on the efficacy of ILPs to improve lifelong learning and best implementation strategies if ILPs are to be most effective.

## Conclusion

In conclusion, our study provides additional evidence that self-assessment and self-directed learning skills are not innately present in physicians; rather these skills are developed over the course of training and development of experience. Incorporation of curricular activities to support development of self-assessment and self-directed learning skills into both undergraduate and graduate medical education is important to the development of lifelong learners. Further study of the efficacy of ILPs and other tools to improve self-assessment and self-directed learning skills is warranted. Effective implementation of ILPs involves faculty development that is focused on fostering and sustaining self-directed learning, and determining the most effective manner of utilizing ILPs. Because lifelong learning is essential to the practice of medicine, medical education curricula must promote the development of self-assessment and self-directed learning skills by integrating the deliberate practice of self-assessment and self-directed learning into residency training.

## Abbreviations

ILP: Individualized Learning Plan

## Competing interests

The authors declare that they have no competing interests.

## Authors' contributions

STL conceived of and designed the study, acquired the data, analyzed and interpreted the data, and drafted the manuscript. MAF participated in the study design, helped analyze and interpret the data, and helped draft the manuscript. DCW participated in interpreting the data and critically revised the manuscript. All authors read and approved the final manuscript.

## Pre-publication history

The pre-publication history for this paper can be accessed here:


